# Salivary *Candida albicans* in asthmatic patients taking anti-asthmatic medication

**DOI:** 10.25122/jml-2022-0142

**Published:** 2022-09

**Authors:** Mohammed Abidullah, Arshiya Sanober, Sandeep Kumar, Kavitha Gaddikeri, Neeharika Soorneedi, Raia Fatema, Safaa Muneer Ahmed

**Affiliations:** 1 Department of Dental & Biomedical Sciences, Faculty of Dentistry, Al Baha University, Al Baha, Saudi Arabia; 2 Department of Oral & Maxillofacial Surgery, Government Dental College & Hospital, Hyderabad, Telangana, India; 3 Department of Public Health Dentistry, Government Dental College & Hospital, Hyderabad, Telangana, India; 4 Department of Oral Pathology, ESIC Dental College, Gulburga, Karnataka, India; 5 Department of Oral Pathology and Microbiology, Malla Reddy Institute of Dental Sciences, Hyderabad, Telangana, India; 6 Aspen Dental, Saint Peters, Missouri, United States of America; 7 Tenet Diagnostics, Banjara Hills, Hyderabad, Telangana, India

**Keywords:** asthma, anti-asthmatic medication, *Candida albicans*, Sabouraud Dextrose Agar

## Abstract

Anti-asthmatic medication makes the oral habitat susceptible to opportunistic infections like *Candida*, causing oral *candidiasis*. This study aimed to estimate salivary *Candida Albicans* in asthmatic patients taking anti-asthmatics medication. A prospective study was performed at the Oral Pathology and Microbiology Department of S.B. Patil Dental College and Hospital, Bidar, Karnataka, India, between June 2018 to November 2018. The research comprised a total of 100 individuals, 50 of whom were asthmatics, and 50 healthy controls who were age and sex-matched to the asthmatics. Saliva was collected for 5–10 minutes in a sterile container, and samples were transferred to the laboratory in cold chain conditions. Serial dilution was prepared for the saliva samples, and 50:1 standard dilution was inoculated on SAD (Sabouraud Dextrose Agar) culture media by lawn culture method. Some part of the culture plate was inoculated with *Candida* organisms. 32 people had *candida* growth, and 18 individuals did not have any *candidal* development at all. 18 people were in the 400 CFU/ml group, and 32 individuals were in the 401 CFU/ml group, respectively. It was 0.000 in the 400 colony forming unit/milliliter group, and 27200 in the 401 CFU/ml group, with 0.00 being the median. There was a notable difference between study and control groups in terms of colony forming unit per milliliter (P=0.000). The growth of *Candida* in asthmatics patients is very high compared to healthy people. Anti-asthmatic medication makes the oral habitat prone to attack from opportunistic infections like oral *candidiasis*.

## INTRODUCTION

Asthma has grown as a significant public health issue in recent years, impacting more than 339 million people all over the world, as per World Health Organization [1]. Over the past decade, the prevalence of pediatric chronic illnesses has increased exponentially in developing countries like India, Africa, and Brazil, making it one of the most prevalent chronic diseases among children, adults and the elderly [2]. Every systemic illness that affects children, particularly during their formative years, has broad consequences [3]. Most of the research has discovered a link between anti-asthmatic medication and the negative impact on oral hygiene, causing decreased salivary flow, xerostomia, difficulty in mastication, dry mucosa, decreased salivary enzymes (IgA, lactoferrin, salivary amylase and lysozyme) resulting in increased dental caries, periodontal damage, in both children and adults with asthma [4]. Dental caries continue to be the powerful cause of tooth erosion in every age group, exacerbating the debilitating effects on both function and appearance [5].

Medications for short-term asthma relief include bronchodilators, anticholinergic drugs and systemic corticosteroids, and long-term control medication includes anti-inflammatory agents, long-acting bronchodilators, leukotriene modifiers and anticholinergic drugs [6]. These medications are mostly administered via inhalation, which may be accomplished using different inhalers or nebulizers [7]. A drug used in these forms will have its effect for a prolonged period, making the oral cavity more inclined to the mucosal insult from these compounds [8]. As in asthma, these medicines, which suppress the immune system and deliver the medicine directly, bring undesirable changes in oral bioflora and support the development of opportunistic infections like *Candida* [9]. *Candida* may grow to unusually high levels in these individuals by the following mechanism:

Neutrophils which are the first line of defense, normally kill about 40% of *candida* growth. However, in asthmatic patients, this activity is diminished along with the reduction in the myeloperoxidase hydrogen halide system due to the immune suppression of the corticosteroids in anti-asthmatic medicines. Other mechanisms of defense diminished are macrophage leukocytes interferon-gamma [10]. These medications significantly reduce local immunity and thus promote the pathogenicity of oral *candidiasis*. In most of these investigations, the levels of *Candida* infection were shown to be inversely linked to the flow of saliva [11, 12].

Therefore, assessing *Candida albicans* (*C. Albicans*) count in response to disease and treatment is very important as this can inform patients about the side effects of medication and emphasize the necessity for oral health care, which will increase the quality of life. This research aimed to evaluate the effect of anti-asthmatic medication on the prevalence of *Candida* in asthmatics patients. This understanding could enable health workers to educate the patients about the side effects of medications and highlight the requirement for oral health care.

## MATERIAL AND METHODS

A prospective study was performed in the Oral Pathology and Microbiology Department at S.B. Patil Dental College and Hospital, Bidar, Karnataka, India, from June 2018 to November 2018. Following the patient's informed consent, a thorough medical history was obtained. The research included 100 individuals, 50 of whom were asthmatics and 50 healthy controls who were age and sex-matched to the asthmatics. The mean age of participants was 35.5 years, with 34 females and 16 males participating in each group.

Inclusion criteria: asthmatic individuals taking medicine for 3–5 years, between 20 to 55 years old.

Exclusion criteria: patients with removable prostheses or who have been taking antibiotics for a long time. In addition, patients who were immunocompromised, smokers, or with chronic diseases were also excluded from the study.

### Collection of samples and microbiological testing

**Figure 1:**
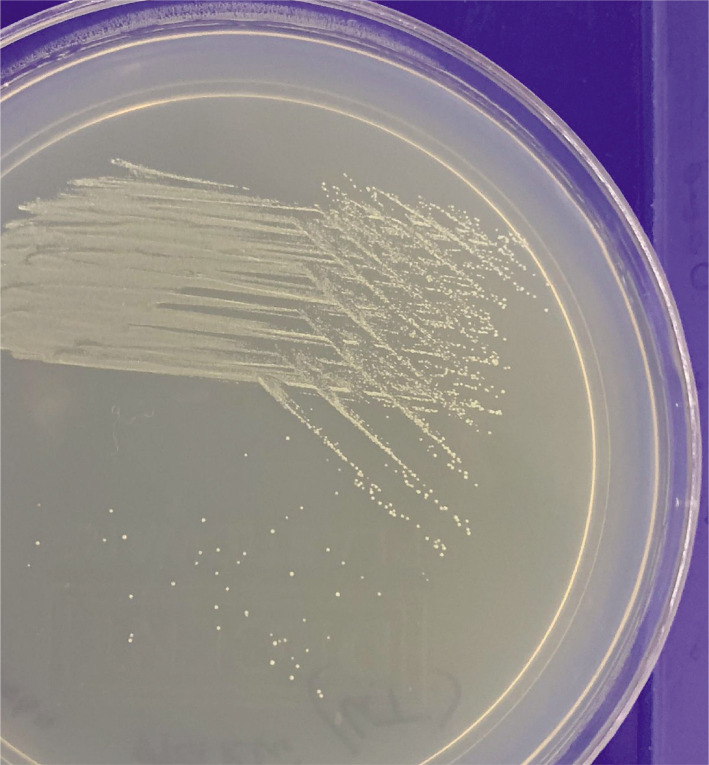
Parts of the culture plate exhibiting growth of Candida organisms.

Saliva was collected for 5–10 minutes in a sterile container, and samples were instantly transferred to the laboratory in cold chain conditions. Serial dilution was prepared for the saliva samples, and 50:1 standard dilution was inoculated on SAD (Sabouraud Dextrose Agar) culture media by lawn culture method. Some parts of the culture plate were inoculated with *Candida* organisms. This plate was incubated at 37.0℃ overnight under an aerobic environment, according to the manufacturer. A morphological examination of *Candida* colonies was performed after incubation (Figure 1), and a germ tube test was performed to establish the presence of *Candida Albicans*. Colony counting was carried out with a computerized colony counter. Colony count was calculated as the number of bacteria (CFU) per milliliter or gram of sample by dividing the number of colonies by the dilution factor. Statistical analysis was done using SPSS version 25.0.

## RESULTS

Patients with 400 CFU/ml were carriers of *Candida*, while cases with >400 CFU/ml were considered pathogenic for *Candida* yeast. Table 1 shows the counts of the salivary *Candida albicans* in CFU/milliliter. 32 people had *candida* growth, whereas 18 individuals did not have any *candidal* development at all. None of the participants in the control group had any signs of *candidal* development.

**Table 1:** The prevalence of *C. Albicans* in both groups.

	*Candida albicans* no growth	*Candida albicans* growth	Total
**Controls**	50	Nil	50
**Cases**	18	32	50

As shown in Table 2, the evaluation of salivary *Candida albicans* counts in CFU/milliliter differed across the patients. A total of 18 people were in the 400 CFU/ml group, and 32 individuals were in the 401 CFU/ml group, respectively. It was 0.000 in the 400 CFU/ml group, and 27200 in the 401 CFU/ml group, with 0.00 being the median. Table 2 shows a notable difference between study and control group in terms of colony forming unit per milliliter (P=0.000). Table 3 shows a notable difference between both groups regarding colony forming unit per milliliter (P=0.000).

**Table 2:** Estimation of *C. Albicans* in the study group.

	N	Median	Mean rank	U	P
**≤400 CFU/ml**	18	0.00	7.29	0.00	0.00
**≥400 CFU/ml**	32	2700	22.69	-	-

**Table 3:** Comparison of *C. Albicans* in both groups.

N	Median	Mean rank	U	P
≤400 CFU/ml (18)	0.00	7.29	0.0	0.00
≥401 CFU/ml (32)	2700	22.69	-	-
Cases (50)	133.0	39.55	122.0	0.00
Control (50)	0.00	20.50	-	-

Table 4 shows the relationship between the duration of the illness and the salivary *Candida albicans* counts in colony forming units per milliliter of saliva. The duration of asthma was split into three groups: three, four, and five years. 19 people (38%) were taking medicine for three years, 19 people (38%) for four years, and 12 people (24%) for five years. There was no statistically significant relationship between duration of illness and colony forming units per milliliter of *Candida albicans* counts (P=0.07).

**Table 4:** Differences between the duration of disease and *Candida Albicans* (CFU/ml).

Duration of disease	CFU/ml ≤400/ml (%)=18	CFU/ml ≥401/ml (%)=32	Total (%)
3 years	6 (31.58)	13 (68.42)	19 (100)
4 years	5 (26.32)	14(73.68)	19 (100)
5 years	7 (58.33)	5 (41.67)	12 (100)

The relationship between doses of anti-asthmatic medicine and salivary *Candida albicans* counts was measured in CFU/ml of saliva. In this research, patients were given anti-asthmatic medicine in doses of 100, 250, or 500 mg, depending on their condition (P=0.17). There was no significant relationship between colony forming unit/ml and antihistamine drugs (Table 5).

**Table 5:** Differences between doses of antihistamine medication and *Candida Albicans* (CFU/ml).

Doses	CFU/ml ≤400/ml (%)=18	CFU/ml ≥401/ml (%)=32	Total (%)
100 mg	9 (40.91)	13 (59.09)	22 (100)
250 mg	8 (40)	12 (60)	20 (100)
500 mg	1 (12.5)	7 (87.5)	8 (100)

Chi-square test, P=0.17.

Table 6 presents the relationship between disease severity and salivary *C. Albicans* counts in CFU/ml. Patients exhibited mild moderate or severe patterns of the disease. There was no significant association between *C. Albicans* count CFU/ml and severity of disease.

**Table 6:** Differences between severity of disease and *C. Albicans*.

Severity of disease	≤400/ml (%)	≥401/ml (%)	Total (%)
Mild	10 (43.48)	13 (56.52)	23 (100)
Moderate	5 (25)	15 (75)	20 (100)
Severe	3 (42.86)	4 (57.14)	7 (100)

Chi-square test, P=0.23.

## DISCUSSION

The presence of *Candida* species in healthy adults with normal salivary gland function has been shown in many investigations. According to several studies, up to 40% of individuals with good dental hygiene have positive *C. Albicans* counts [13].

*C. Albicans* is a very common infectious organism in asthma patients. Nebulization is the best method for delivering the drugs, and we can compare the colony forming unit per milliliter in adult patients in the same age group in which nebulization was not used [7].

50 people diagnosed with asthma who were taking medication and met the previously stated criteria and 50 healthy matched individuals were included in this study. The CFU/ml of *Candida albicans* in saliva was determined in the patients and controls using conventional techniques on Saliva Diagnostic Assay. Thirty-two (64%) of the participants had *candida* growth, whereas 18 (36%) had no *candidal* development, suggesting that asthmatics on medication had a microfloral change that facilitated *candidal* growth compared to healthy persons in the study. The colony forming unit was measured in asthmatics patients, and a 1000 medium value was obtained.

A remarkable difference was found between the case and control groups. As a result, among asthmatics with *candida* growth, there were 32 people with salivary *Candida* counts of ≥401 CFU/ml (≥401 CFU/ml were considered pathogenic) [14, 15]. All of the results above suggest that the prevalence of *Candida albicans* among asthmatics who use anti-asthmatic medicines such as corticosteroids and β2 agonists has risen. In addition, asthmatics had a colony forming unit count comparable to that of infective *candidiasis, i.e*., 401 CFU/ml of saliva, while healthy individuals did not.

It is worth noting that, despite the high CFU counts, which suggest *candidal* infection, none of the patients presented with symptoms of oral *candidiasis* throughout the study. This may indicate that the infection was subclinical or microbiotic shift occurred, allowing greater *candidal* development to occur without causing symptoms. Several comparable studies have been conducted, all of which suggest and corroborate the findings mentioned above that asthmatics who use inhaled anti-asthmatic medication are more susceptible to *candida* infection, and treatment is necessary to improve the quality of life [16, 17].

This discovery has been interpreted differently, including the interaction between illness, medicine, and the host [18]. *Candidal* development is facilitated by bronchial asthma, and patients with asthma who have restricted salivary flow are more prone to mouth breathing [19]. Asthmatics who breathe via their mouths for extended periods develop xerostomia and a changed microbial ecology in their oral cavity, which increases the growth of *Candida* organisms. Inhibition of the host factor by anti-asthmatic medication results in increased *candida* growth. The control of the asthmatic condition is achieved primarily through medications that fall into two categories: β2-agonists (β2-agonists), which promote bronchial relaxation, and corticosteroids, which suppress the immune system [20].

In contrast to previous studies, the current research found less evidence of *candidal* carriage in healthy people. Cohen et al. [21] discovered that yeast was present in the oropharynx of healthy participants at a rate of 35%. In their research, Hanan et al. [22] discovered that oral *candidiasis* occurs at a rate of 30 to 45% in healthy individuals. According to Zaremba et al. [23], who performed a research on the oral carriage of *Candida* in healthy people, the prevalence rate was 63.1%. Healthy individuals with *C. Albicans* in their oral cavity were approximately 3–48% of the total population [24]. In addition to geographic differences, the type and size of the sample chosen and the technique of sample collection are factors that should be addressed. Examples of such variables include the sample size, the individual tested, the sampling technique used, and the estimates of *Candida* species prevalence as human commensals may differ significantly [25, 26].

Regarding asthmatic patients, the researchers looked at the relationship between the length of asthma, the severity of illness, and drug dose. There was no statistically significant relationship between associated factors and the *C. Albicans* population. Asthmatic patients are more prone to *candidal* infection, the seriousness of diseases, and drug dosage, and it is viable that medications expand the patient's susceptibility to *candida* growth.

## CONCLUSION

The growth of *Candida* in asthmatics patients is very high compared to healthy people. Anti-asthmatic medication makes the oral habitat prone to attack from opportunistic infections like *Candida* causing oral *candidiasis*. Therefore, assessing *C. Albicans* count in response to disease and its treatment is mandatory. This understanding enables health workers to draw strategies to educate the patients about the side effects of medication and highlight the requirement for oral health care. More studies with a larger sample size are needed as such research throws light on the preventive measures to be adopted, such as orienting the patients for frequently mouth rinsing, using spacer devices, promoting topical antimycotics (nystatin) application, using sialagogue medication in patients with low salivary rate, chewing sugar-free gums, gargling with amphotericin diluted 1:50 solution, low cariogenic diet and advocating less dosage ICS.
